# Meaning-making in home-based end-of-life care: a qualitative study of wives’ experiences after the loss of their husbands to cancer

**DOI:** 10.1186/s12904-026-02095-z

**Published:** 2026-05-12

**Authors:** Mari Karikawa, Hisae Nakatani

**Affiliations:** 1 Department of Nursing, Faculty of Health and Welfare, Prefectural University of Hiroshima, 1-1, Gakuen-Chou, Mihara, Hiroshima 723-0053 Japan; 2 Graduate School of Biomedical & Health Sciences, Hiroshima University, 1-2-3 Kasumi, Minami-Ku, Hiroshima, 734-8551 Japan

**Keywords:** Bereaved spouses, Patients with terminal cancer, Qualitative longitudinal study, Spousal caregiving, Meaning-making

## Abstract

**Background:**

Caring for a spouse with cancer at home during the end-of-life period is emotionally demanding and can have lasting effects on bereaved partners. For some wives who assume the primary caregiving role, this experience becomes a source of meaning, personal growth, and relational reconstruction after loss. However, the longitudinal processes through which they derive positive meaning and rebuild their relationship over time remain unclear. This study builds on our previous research involving bereaved wives and husbands, who provided home-based end-of-life care for a partner with cancer between 6 months and 2 years after bereavement, and examines their positive interpretations of caregiving. In this follow-up study conducted approximately two years later, we re-interviewed the wives to explore how they interpreted their caregiving experiences. Through longitudinal comparison with the initial findings, we examined how caregiving meanings evolved.

**Methods:**

We used a qualitative longitudinal design and conducted second interviews (approximately four years after bereavement) with wives who participated in the initial study and provided renewed consent. The semi-structured interviews were transcribed verbatim and analyzed using sequential comparative analysis.

**Results:**

Five themes were identified: “the enduring bond of the married couple,” “the importance of family members who spent time together until the end,” “the meaning attached to caring for the husband in the terminal phase,” “confronting life without the husband,” and “reflection on one’s way of life.” Over time, wives gradually reconfigured their relationships with their husbands, shifting from viewing caregiving as a “concluded past” to experiencing it as a “continuing relationship in the present.” This transition represents a conceptual shift, illustrating a dynamic process in which earlier interpretations are revisited and integrated into an evolving life narrative.

**Conclusions:**

The meaning-making process surrounding care for a dying husband did not remain fixed after bereavement; it evolved as a dynamic, ongoing reinterpretation. The findings indicate that positive meaning-making after loss deepens over time and is accompanied by relational growth. Through end-of-life care support, visiting nurses can understand how wives “make meaning of their marital relationship” and provide opportunities for narrative exchanges that lead to post-bereavement meaning construction.

**Supplementary Information:**

The online version contains supplementary material available at 10.1186/s12904-026-02095-z.

## Background

In Japan, over 380,000 people are diagnosed with cancer and die each year [1]. Since 1981, cancer has been the leading cause of death nationwide; as the population ages, the number of deaths is expected to continue to increase. Inpatient care for patients with cancer is becoming increasingly difficult, and the treatment and care environment for terminally ill patients with cancer is shifting from hospitals to home-based care. Japan’s Ministry of Health, Labour and Welfare has identified strengthening the coordination between medical and nursing care systems and enhancing home-based medical care as key policy measures [2], and institutional reforms aligned with these goals have been implemented.

Home-based palliative care places significant responsibilities on family caregivers [3]. Assuming the role of a family caregiver can constitute a negative life event and potentially disrupt many aspects of the caregiver’s existence [4]. Feelings of helplessness and guilt experienced by family caregivers owing to their perceived inability to help the patient with cancer [5], alongside caregiving burdens [6], lead to depressive symptoms. Psychological distress among family caregivers of terminally ill patients with cancer persists even after the patient’s death. Among the family members of patients with cancer receiving hospice home care, over 50% exhibited clinically significant depressive symptoms 1 year after bereavement [7].

Longitudinal post-bereavement studies have focused primarily on changes in depressive symptoms and grief intensity, demonstrating diverse patterns of grief reduction and resilience over time [8, 9]. Many of these studies viewed grief primarily through pathological and recovery-oriented lenses, focusing on reducing depression and psychological distress. These studies did not adequately address the dynamic processes that occur over time after bereavement, such as the construction of positive meaning and relationship reorganization.

Neimeyer [10] proposed the concept of “meaning reconstruction” to address how individuals who experience bereavement accept and understand their loss. The concept describes a dynamic process in which an individual reorganizes their life narrative through loss and reconstructs the meaning of events temporally [11, 12]. Based on this perspective, psychological adaptation after bereavement is reframed not as the “disappearance of grief” but as the “reconstruction of meaning.” Furthermore, recent grief research has focused on the concept of “continuing bonds,” where individuals maintain connections with the deceased even after loss [13]. This framework proposes understanding loss not as an “end” but as a “transformation of relationships” [14]. Moreover, in Japan, many bereaved family members describe a continued sense of the deceased’s presence in daily life and a tangible feeling that the relationship endures even after death [15].

Many of these theoretical frameworks focus on reconstituting meaning and continuing bonds triggered by bereavement. However, only a few longitudinal studies have clarified the subsequent changes in the meaning formed through the temporal and relational process of the “caregiving experience” prior to bereavement.

Spouses who have lost a partner to cancer experience depressive symptoms at rates two to three times higher than those of other family members [16]. Studies focusing on wives who lost their husbands to cancer report markedly higher levels of physical and psychological symptoms—including depression, insomnia, fatigue, and loss of appetite—than those of other family members [17, 18]. They also tended to experience prolonged grief and loneliness after bereavement [7, 18]. However, the experiences of wives who cared for their spouses at home until death are narrated within a temporal continuum encompassing “caregiving,” “bereavement,” and “life after loss.” Within this narrative, the “pride of having fully supported her husband as a wife” and the “sense of loss from losing her husband” are intricately intertwined with the loss of the “role consciousness of the wife supporting the family” that is cultivated in the Japanese culture. We believe that understanding the change in meaning within this context—not merely as a psychological recovery process but also as relational growth and a reconfiguration of role consciousness rooted in cultural values—can improve our understanding of loss within Japan’s cultural context.

This longitudinal study, therefore, aimed to clarify the process of reconfiguring the meaning of the end-of-life care experience over time, focusing on wives who had positively reframed their caregiving experience after providing home-based care for a spouse with cancer and were between six months and two years after the loss.

## Methods

### Sample and recruitment

The first survey [19] of our study involved interviews with 13 individuals (two men and 11 women) who had cared for and witnessed the death of a spouse with cancer at home. Based on a qualitative analysis of these interviews, participants were classified according to the degree of “positive meaning-making” related to their caregiving experience. Individuals who struggled to accept the death of their spouse and were unable to derive positive meaning from the caregiving process were excluded from the follow-up study. Participants who accepted the death of their spouse and expressed gratitude or a sense of fulfillment in the relationship were defined as demonstrating positive meaning-making (*n* = 11; two men and nine women). In the initial study, both wives and husbands were interviewed to explore positive meaning-making following home-based end-of-life caregiving. Although husbands described their caregiving experiences in meaningful ways, their narratives tended to emphasize time management, role-based responsibility, and practical adaptation to caregiving demands. In contrast, wives provided more detailed and conceptually rich accounts concerning relational continuity, marital identity, and the positive reinterpretation of caregiving within an ongoing life narrative. Given the exploratory nature of the initial study, the follow-up phase was designed to further examine and refine this emerging relational meaning-making process. Therefore, the present longitudinal analysis focused on wives in order to deepen understanding of the temporal reconfiguration of caregiving meaning within a more homogeneous and theoretically coherent sample. This analytic focus was intended to enhance conceptual clarity and processual depth rather than to negate the relevance of husbands’ experiences. Such analytic narrowing is consistent with qualitative longitudinal research, in which later phases may focus more closely on theoretically salient processes identified in earlier analyses [20].

**Fig. 1:**
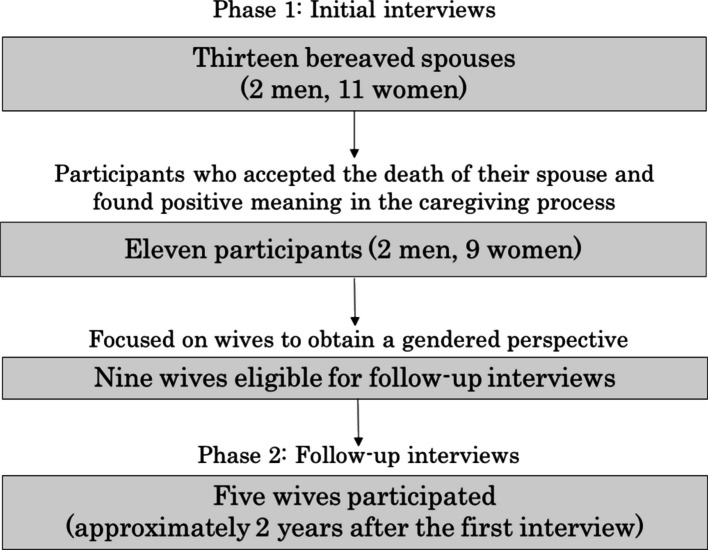
Sampling flow and the participant selection process

Of the nine eligible wives, five agreed to participate in the second interview. The remaining four declined due to health conditions or concerns about the psychological burden of revisiting their experiences (Fig. 1). Each of the five participants engaged in two in-depth interviews, each lasting approximately 1–2 h, which generated rich narrative data across two time points. The longitudinal design enabled detailed within-case analysis of meaning shifts, as well as cross-case comparison of the continuity, emergence, and reorganization of themes. In qualitative longitudinal research, the adequacy of sample size is evaluated in terms of information power rather than numerical representativeness [21]. Information power increases when the research aim is narrow, the sample is specific, the data are rich, and the analytic strategy is in-depth. In addition, longitudinal designs enhance information power by providing temporal depth, thereby allowing researchers to capture evolving interpretations and processes of change over time within cases [22]. The present study had a clearly defined aim—to explore longitudinal meaning reconfiguration among bereaved wives who had previously demonstrated positive meaning-making. Given the focused research question, depth of data, and analytic emphasis on temporal processes rather than population variability, information power was considered sufficient to support this study’s findings.

The first author has professional experience in home-based nursing care but was neither employed by nor directly affiliated with the participating visiting nurse stations at the time of the study. However, familiarity with the context of home-based end-of-life care may have influenced both data collection and interpretation, and this positionality was continuously examined through reflexive practices, including memo writing, maintenance of an audit trail, and peer debriefing. Participants were recruited through purposive sampling, with visiting nurse station managers serving as gatekeepers. Managers were provided with written inclusion criteria and asked to identify all individuals who met these criteria rather than selectively approaching potential participants based on perceived willingness or narrative characteristics. In requesting recruitment assistance, particular attention was paid to ethical considerations. Managers were informed that discussing bereavement experiences might impose a psychological burden on potential participants. Consequently, they were asked to approach eligible individuals sensitively and ensure that participation was entirely voluntary. In addition, each visiting nurse station was instructed to designate a contact person within the organization so that participants could consult with a familiar nurse if they experienced emotional discomfort related to study participation. This arrangement was established to ensure participants’ psychological safety and mitigate potential harm. After initial contact, all communication regarding participation occurred directly between the researcher and potential participants. To reduce potential organizational pressure, nurse station staff were not informed about who agreed or declined to participate. Furthermore, several strategies were employed to minimize potential bias related to recruitment and interpretation. An audit trail documenting recruitment decisions, interview procedures, coding processes, and theme development was maintained. Reflexive memos were written throughout data collection and analysis to examine the potential influence of the researcher’s professional background and assumptions on data interpretation. Additionally, peer debriefing was conducted within the research team to critically review emerging themes and challenge potential confirmation bias.

### Data collection

Semi-structured interviews were conducted with the research participants in their homes to ensure privacy. The first interview with 13 participants, conducted six months to two years after bereavement, included the following items: 1) the condition of the patient receiving home care and the caregiving situation; 2) the participant’s daily life during caregiving; 3) the participant’s feelings and thoughts during patient care; and 4) their reflections on the meaning and value of their home caregiving experience.

Subsequently, we conducted a second interview of spouses from among the five participants who had attributed positive meaning to their end-of-life care experience during the first interview and agreed to further interviews. During the second interview, participants were reminded of selected statements or thematic summaries derived from their first interviews. This approach was employed to facilitate reflective comparison in line with qualitative longitudinal methodology. Supplementary Table 1 provides detailed information on the interviews. To mitigate potential leading effects, neutral and open-ended prompts were employed, such as “How do you feel about this now?,” “Would you describe this differently today?,” and “Does this still represent your experience, or has your understanding changed?”.

Participants were explicitly informed that they were free to revise, expand, or disagree with their previous statements. In practice, several participants modified their earlier interpretations, revealed new perspectives, or shifted emphasis, indicating that the reminder appeared to function as a catalyst for reflection rather than as a directive constraint.

The interview content was recorded with the permission of the research participants. The second interview was conducted between May and August 2015, two years after the first interview. A two-year interval was selected to capture later-phase changes in bereaved spouses’ meaning-making in terms of their caregiving experience.

Meaning reconstruction theory [10] and the continuing bonds framework [13] suggest that integrating loss into one’s life narrative progresses over an extended period. In addition, longitudinal bereavement studies have reported that significant psychological and relational changes often occur between 18 and 48 months post-loss [8, 23]. Therefore, a two-year interval was deemed appropriate for capturing these developmental processes while minimizing recall burden for participants. Each interview lasted approximately one–two hours, and each participant was interviewed once.

### Analysis

This study employed a qualitative longitudinal research design to explore temporal changes in meaning-making following spousal bereavement. Data were analyzed using reflexive thematic analysis informed by a longitudinal comparative framework. To clarify how the meanings ascribed to caregiving and end-of-life experience change over time, we conducted an initial set of interviews with wives who had provided home-based end-of-life care for their husbands, at six months to two years after bereavement [19]. Approximately two years later, we conducted a second set of interviews with the same participants. This interval was chosen to capture a period in which initial grief responses generally subside and deeper processes of meaning reconstruction are thought to occur [10].

The analysis involved three stages. First, the second-interview transcripts were inductively analyzed to generate themes reflecting participants’ current interpretations. Second, within-case longitudinal comparison was conducted by examining each participant’s first and second interviews to identify continuities, shifts in thematic salience, and emergent meanings. Third, across-case comparison was performed to identify common longitudinal patterns across participants.

Thereafter, we created a longitudinal comparison matrix contrasting the themes in the first and second interviews to identify patterns of continuity and change in the narratives. Through this process, we identified newly emerging meanings or meanings that persisted temporally, extracting the process of “meaning reconstruction” across both time points. Theoretically, this comparative analysis positioned the process of narrative reconstruction over time by referring to Gillies and Neimeyer's [11] theory of meaning reconstitution and Park’s [12] concept of integrative re-meaning.

The authors independently conducted coding to ensure the reliability of the analysis. After theme extraction, the researchers held repeated discussions to reach a consensus. Throughout the analysis, the researchers ensured reliability and validity through ongoing supervision provided by two community and home nursing specialists. Furthermore, bilingual researchers verified translations to preserve cultural and linguistic nuances.

### Ethical considerations

We asked the managers of the visiting nursing stations who had assisted with a previous study to contact former participants and invite them to take part in the present study. The researchers explained the study’s purpose and ethical considerations to the managers both verbally and in writing. The managers then contacted potential research participants as a part of bereavement support, asking for their consent to send them a document outlining the study’s purpose and ethical considerations. After receiving consent, the researchers sent the materials to those individuals. If they agreed to receive a full explanation of the study, the researchers arranged a meeting with them subsequently. During the meetings, the study purpose and ethical considerations were explained both verbally and in writing. The study was conducted after consent for participation was obtained. This study was approved by the Ethics Review Committee of Kobe City College of Nursing.

## Results

### Sample

The study participants included five wives who had cared for their husbands with cancer and experienced bereavement at home. Participants were between their 30 s and 70 s. The period from the diagnosis of a terminal illness to patient death was 20–180 days. The duration of home care was 20–90 days. The time that elapsed between bereavement and the first interview was 12–24 months. The second interview was conducted two years after the first (Table 1).

**Table 1:** Overview of the study participants

Research Participants	Relationship with the Patient	Age	Diagnosis	Period from the diagnosis of a terminal illness to death	Period requiring care	Period from bereavement to first interview	Period from bereavement to second interview
A	Wife	70 s	Lung cancer	4 months	3 months	19 months	38 months
B	Wife	30 s	Brain tumor	2.5 months	1.5 months	13 months	33 months
C	Wife	70 s	Liver cancer, oropharyngeal cancer	6 months	2 months	21 months	41 months
D	Wife	50 s	Bile duct cancer	5 months	1 month	24 months	46 months
E	Wife	60 s	Stomach cancer, prostate cancer	20 days	20 days	12 months	31 months

The six themes identified in the first interview centered on marital relationships, relationships with family and others, caregiving experiences, self-existence, and views on life and death. In the second interview, themes such as the enduring bond of the married couple, the importance of family members who spent time together until the end, the meaning attached to caring for the husband in the terminal phase, confronting life without the husband, and reflection on one's way of life were extracted. These results indicated continuity in many of the major themes extracted at both time points. However, over time, the focus shifted from external relationships to the participant’s inner world (Table 2). These findings also suggest patterns of dynamic and integrative meaning reconfiguration among wives who had previously constructed relatively positive interpretations of their end-of-life caregiving experiences. Below, for each theme, we present narratives from the second interview and relate them to the first interview’s results.

**Table 2:** Comparison of key themes at two time points

Primary themes in the first interview (6–24 months after bereavement)	Key themes in the second interview (approximately 2 years later)	Characteristics of longitudinal relationships and changes
The profound significance of existing as a couple	The Enduring Bond of the Married Couple	The meaning of being a couple was reconfigured over time as a shared existence that continued to be lived together, deepening into an inner bond
Awareness of an unshakable family relationship	The Importance of Family Members Who Spent Time Together Until the End	Narratives about family bonds are consistent, shifting from family as a source of support to family as a foundation for self-identity
Presence of Surrounding Support	(Did not appear as an independent theme in the second round)	Although the significance of surrounding support was mentioned, it weakened as a primary theme, shifting focus to the inner world of family and spouses
Realizing the ideal end	The Meaning Attached to Caring for one’s Husband in the Terminal Phase	Although satisfied with having achieved an ideal farewell, the participant felt self-reproach, such as whether they had done enough for their husband. The participant was reframing the caregiving experience itself as proof of love and growth
(Did not emerge as an independent theme in the first session)	Confronting Life Without One’s Husband	Although not seen as an independent theme in the first session, in the second session, by sharing the caregiving experience with others, the participant retained the husband’s presence in the heart and developed a new challenge: how to live life without the husband
Confidence in one’s existence Deepening views on life and death	Reflection on One’s Way of Life	Over time, meaning frameworks concerning self and life–death issues deepened to the point that the participants used them to reconstruct their lives and integrated them into their way of living

This table presents the results of a longitudinal qualitative analysis of 13 spouses who cared for patients with cancer until the death of the patients (including five participants in the second follow-up)

Each theme was generated according to the narratives of all participants at each time point, and the relationships between the two time points were organized through content analysis and re-examination of the narratives.

### The enduring bond of the married couple

In this theme, the wife remained content with the peaceful daily life shared with the husband until death, even with the husband’s diminished abilities and increased care needs. Even after the husband’s death, the spouse felt the presence of the husband and remained conscious of their marital bond. In the second interview, participants spoke of their bond with their husbands not as something “lost” but as something “still present in their lives.” This theme, identified in the first interview as “the profound significance of existing as a couple,” gained deeper significance through the experience of spending time together until the very end, with the wife realizing that her partner had been thinking of her until his final moments.

“Before he died, he left me a note. I was so touched that they took the time to write this—it’s truly a treasure to me now. It said, ‘I can't stop crying as I say these words of gratitude.’ I think they must have been really happy. I believe those tears were tears of gratitude toward me. Reading each and every word, I was so happy to think he’d left me so many words.” (Ms. D) [19]

However, the second interview revealed that, over time, the theme shifted to the continuation of the marital relationship as it transformed in form and the reconfiguration of its meaning.

Two subthemes emerged from this: *“a sense of fulfillment in the final days of daily life spent consciously as a couple”* and “*the sense of remaining connected to one's husband*.”

#### A sense of fulfillment in the final days of daily life spent consciously as a couple

This subtheme indicates that the wives felt satisfied in maintaining a relationship in which they could exchange jokes until the very end and in the ability to share time and space without feeling self-conscious. These wives found joy in preserving their lives together until the end, even while being conscious of their husbands’ imminent death. One wife shared the following:

When I went out shopping, he worried about me. When he was asleep, I felt reassured and would quietly go check on him. If he was awake, we'd have tea together. I truly feel so glad I was able to be at home. (Ms. A).

#### The sense of remaining connected to one's husband

The wives cherished memories of their husbands’ final days, feeling their presence close even after death, as they continued with their lives. One wife stated as follows:

In the end, it's the living who matter. So, if you can make the departed person your anchor in your heart, I think that's what it means to live together. (Ms. B)

While the bond itself remained continuous, the narratives from the second interviews suggest that a shift occurred from retrospective gratitude toward an internalized and enduring presence integrated into everyday life.

### The importance of family members who spent time together until the end

This theme reflects the family’s shared experience of caring for a husband whose health was steadily declining and supporting him at home until the end of life. It also shows that continuing to share this experience as a family provides an opportunity to reaffirm the value of their bonds and their collective sense of identity. This theme yielded two subthemes: “*the sense of unity with family members who shared one’s husband's final moments*” and “*the family connection alive in everyday life*.”

#### The sense of unity with family members who shared one’s husband's final moments

Wives described the time spent caring for their husbands and their families as a precious period of shared life. Being united in supporting the husband until death deepened family bonds, and this shared experience continued to serve as a lasting emotional connection. One wife shared the following:

Since my children and grandchildren could visit only for a few days, I don't think we ever had this much time together as a family. So, I suppose the greatest advantage of caring for him at home is that everyone could support my husband with the same heartfelt feelings and that the months we spent together would remain as something profound in our hearts afterward. (Ms. D)

#### The family connection alive in everyday life

The wives continued to feel connected to their husbands through their ongoing relationships with family members. The bonds formed while caring together for the dying husband remained present in their lives and emotions long after bereavement. For wives, this served not only as an emotional connection but also as support in their future lives. One wife shared the following:

My husband relied heavily on our son. I think it was good that it wasn’t just me alone; my son and I cared for him together until the end. Even now, my son comes to my house every night. He lights incense for his father and spends 10 or 15 minutes talking to him about work, just like he did before he passed away. My son’s presence means so much to me. I rely on him for everything. Just having him there, that presence, is what matters. (Ms. C)

Participants reflected not only on shared memories of their husbands’ final moments but also on how those experiences had become woven into their ongoing relationships. In the first interviews [19], participants also noted that caring for and staying close to their husbands until the end deepened their appreciation for family ties.

“I treasure the unity we experienced as a family over those five condensed months while my husband was receiving care at home.”

Here, family was primarily seen as a source of strength and solidarity during the caregiving period. Compared to the initial narratives in which family unity was described primarily as a coping resource during caregiving, the follow-up interviews positioned family bonds as an enduring relational foundation that continues to shape the participants’ present lives. This shift suggests a movement from situational solidarity to temporally extended connectedness embedded in everyday life. This temporal extension of family meaning illustrates how shared end-of-life experiences are not only remembered but are also gradually integrated into ongoing relational life.

### Presence of surrounding support

In the initial interviews conducted 6–24 months post-bereavement [19], participants frequently emphasized the importance of surrounding individuals who supported them during their husbands’ end-of-life period. For example, one participant stated, “There’s a friend who used to come to our house every week, and we still keep in touch. I’m so grateful. Not only did they care about my husband, but they’ve continued to look out for me even after he passed away.” Such narratives positioned external support as a central element of affirming the value of the end-of-life experience.

In contrast, in the follow-up interviews, references to surrounding support were less prominent. While participants still acknowledged the presence of others, their narratives included more personal and existential reflections. The emphasis moved from describing external assistance to articulating internal processes, such as confronting absence and reconsidering one’s way of living.

This relative reduction in the salience of surrounding support became apparent only through longitudinal comparison and suggests a gradual re-centering of meaning from external relational affirmation toward inner narrative integration.

### The meaning attached to caring for one’s husband in the terminal phase

This theme captured the care that the wives provided during their husbands’ final days and illustrated how they derived meaning in supporting them at home with help from others. In the second interview, some participants expressed regret about conversations they wished they had had and offered a more reflective reassessment of their caregiving experience.

This theme comprised three subthemes: “*pride in supporting him as his wife until the end*,” “*gratitude for the help provided by professionals*,” and “*regrets about how to spend the last moments with one’s terminally ill husband*.”

#### Pride in supporting him as his wife until the end

Wives felt pride in having supported their husbands throughout their lives. They spoke of providing the care they believed their husbands desired—providing support in whatever they wished to do and staying close to observe the slightest change in their condition—and witnessing their husbands’ peaceful death. Two wives shared the following:

Of course, being woken up in the middle of the night was somewhat difficult. When my husband said, “I’m hungry,” at night, I made everything right here (in the kitchen). Had he been in the hospital, even if he hadn't called my name, if he had said “I want some water” in the middle of the night, I'd have had to call a nurse. All those things were possible because it was me and because we were here (at home). I slept right here (in the living room) with him. I could see his face all the time, and I truly believe that was absolutely best for my husband. Maybe it's just my own self-satisfaction.” (Ms. A)

At the time, I was desperate, but after he passed away, I felt, “Ah, I'm so glad.” Back then, when I watched professionals change his diapers, I thought, “Oh, this is how you change a diaper.” But I never considered leaving him alone to do something else. I felt I had to stay by his side the whole time. (Ms. C)

#### Gratitude for the help provided by professionals

Even with the imminent death of their husbands, the wives felt a deep sense of gratitude for the presence of doctors and nurses who allowed them to peacefully approach end-of-life care and fostered relationships that allowed open communication. One wife shared the following:

Seeing my husband chatting with the doctor about daily hobbies and events, I thought he looked so happy. Even when he was bathing, the nurse would wash his back while they talked about all sorts of things. I was glad he could do that, and I felt happy. (Ms. A)

#### Regrets about how to spend the last moments with one’s terminally ill husband

The wives felt regret because they were aware of their husbands’ imminent death but avoided discussing it or expressing their true feelings regarding this situation. Additionally, they regretted being overly harsh with their husbands during end-of-life care because of feeling overwhelmed and experienced self-reproach about whether they provided sufficient support.

For example, 33 months after the husband’s death, Ms. B regretted not having shared their mutual wishes about how they would spend their final days. Ms. B said:

I really didn’t want to talk about death. I didn’t want to face it myself, and I didn’t want my husband to sense that either. As he approached death, maybe he had more wishes about what he wanted, but I never created that kind of atmosphere at all. In a way, I think I blocked it off myself. (Ms. B)

In the initial interviews [19], caregiving was primarily described in positive terms, and wives expressed no regrets about their caregiving.

“If he had been in the hospital, he’d be hooked up to monitors and IV drips. I think it’s better that he was able to die naturally than if he had died in one of those places.” (Ms. C)

This shift suggests not a reversal of meaning but rather the emergence of reflective ambivalence and a gradual refinement of previously positive interpretations.

### Confronting life without one’s husband

This theme captured how bereaved women navigated their daily lives with the absence of their husbands, who had once supported them, as well as how they confronted the emotions that surfaced in that absence. The derived subthemes were *“the sadness of life without one’s husband”* and *“sorting out one’s feelings.”* This theme did not emerge as an independent category from the earlier interviews conducted 6–24 months post-bereavement [19]. In the initial narratives, participants primarily emphasized the meaningfulness of caregiving and affirmation of relational bonds. In contrast, in the follow-up interviews, greater attention was directed toward the daily reality of life without the husband. The temporal distance from bereavement appeared to create space for acknowledging the enduring nature of absence; it is not only a memory of loss but also an ongoing life condition. Rather than simply recalling the past, participants described the practical and emotional work of reorganizing daily life and processing complex feelings. This shift, which became discernible only through longitudinal comparison, suggests a gradual reorientation from reflecting on the caregiving experience itself toward confronting the continuing implications of living without one’s spouse.

#### The sadness of life without one’s husband

Wives recalled their husbands’ presence, sometimes feeling a surge of loneliness, regretting that they could not spend the rest of their time together, and experiencing a sense of sadness. One wife shared the following:

I suppose you do get used to life without him, but you never get used to the loneliness. Being alone, that loneliness never changes, no matter how much time passes. I often think, “If only he were here.” (Ms. D)

#### Sorting out one’s feelings

By sharing their caregiving experiences with others, the wives could objectively reflect on their feelings. Moreover, they found that people acknowledging that their loved one died at home helped them manage their feelings. One wife stated the following:

By talking about how I felt when I was caring for my husband, I feel like I could make better sense of my emotions. When I think back to what I said in the first interview, I realize that’s how I truly felt at the time. Comparing that to how I feel now, I see there’s a bit of a difference. It makes me realize that going through that experience shaped who I am today. (Ms. B)

### Reflection on one’s way of life

This theme reflected bereaved wives’ thoughts on life and death, which deepened through caring for their terminally ill husbands, and their considerations for how they would live their lives in the future. It consisted of three subthemes: *“living with the legacy of one’s husband’s final kindness and wishes in one’s heart,” “ contributing to others by applying end-of-life care experience,”* and* “the views of life and death learned from one’s husband’s final moments of life.”* In the first interview, participants expressed their thoughts about how to live their lives, but these were often expressed in terms of realizing their dying husband’s wishes or choosing a lifestyle that would not burden those around them. In the second interview, however, based on this understanding, they reflected on their husbands’ way of life in the final moments and reinterpreted the feelings their husbands had about their own lives in a way that would support their present lives. Furthermore, based on this experience, they began to think more concretely about not only how they would live their future lives but also how they would face death.

#### Living with the legacy of one’s husband’s final kindness and wishes in one’s heart

Two wives stated:

My husband, in his terminal phase, was conscious of his impending death. He reflected on the life his wife would live after he was gone, leaving behind letters, words, and memories that became her life sustenance.

He wrote (in a letter before passing) that since I was always a cheerful person who could make lots of friends, I should go out into the world. That made me think, “Oh, right.” I believe I should find and do enjoyable things from now on. My husband wrote that I should do just that. (Ms. C)

#### Contributing to others by applying end-of-life care experience

By caring for their husbands, the wives shared their caregiving experiences with others and helped them meet their needs. These actions allowed them to utilize their past caregiving knowledge to assist others. Two of the wives shared the following:

Elderly members still come to church in good health. Of course, as they age, they come with canes, but even when they take their Bibles to their seats, I don’t mean to say I’m nursing them, but I’ll hold their cane for them and say, “Take my hand,” and walk with them. It might be an exaggeration to call it sincerity, but perhaps because I was nursing him, it just comes naturally. (Ms. A)

Having my sister and her husband by my side as we cared for my husband in his final days gave me great strength. That’s why, if any of my other siblings ever need help, I’ll be there in a flash. (Ms. E)

#### The view of life and death learned from one’s husband's final moments of life

Wives who witnessed their husbands’ final moments while providing care described searching for ways to live without burdening their families, pursuing fulfillment in their own lives, and recognizing the preciousness of life. Simultaneously, they began to contemplate their own deaths. Experiencing death as closely as life prompted them to re-examine their views on both. The wives shared as follows:

My husband said he would insert his own feeding tube and seemed convinced it was necessary, but watching him, I wonder if it’s really needed. I want him to stay with me. But I don’t think it’s necessary to extend someone’s life that far. If I ever need a feeding tube, I won’t have one inserted. I think if I can’t eat, it’s fine to just let things take their natural course. (Ms. C)

Dying at home isn’t a death filled with suffering and agony; it’s a peaceful way to go. That’s why I want to live my ordinary life at home, growing weaker little by little. (Ms. D)

Toward the end, I laid our grandchild next to my husband, and I slept beside them. We were exhausted too, and while we dozed off, he passed away. So, I think he left peacefully. He absolutely hated the idea of being bedridden forever. That’s why I think it was a death with dignity. He said he didn’t want to live if it meant losing his dignity. (Ms. D)

## Discussion

This study examined the longitudinal patterns among bereaved wives who had previously demonstrated positive meaning-making, focusing on how spouses of patients with cancer who had positively reframed their end-of-life care experiences 6–24 months after bereavement reconceptualized those experiences two years later. As the sample intentionally comprised wives who had previously articulated relatively affirmative interpretations of caregiving, the findings reflect patterns within this particular subgroup rather than the full spectrum of bereavement experiences.

The results revealed five themes: the enduring bond of the married couple, the importance of family members who spent time together until the end, the meaning attached to caring for one’s husband in the terminal phase, confronting life without one’s husband, and reflection on one’s way of life. Although these themes largely aligned with findings from the initial interview conducted six months to two years post-bereavement [19], important temporal developments became evident through a longitudinal comparison.

**Fig. 2:**
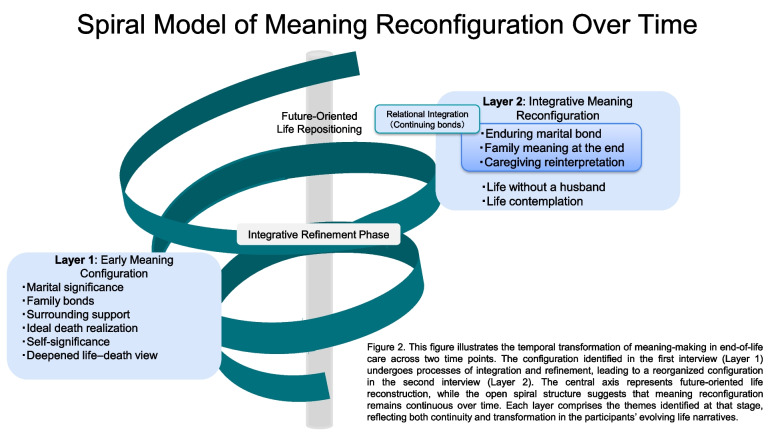
Spiral model of meaning reconfiguration over time

Notably, “confronting life without one’s husband” emerged as a new theme that had not been identified in the initial cross-sectional analysis. In addition, within the theme concerning the meaning of caregiving in the terminal phase, a new subtheme emerged-* “regrets about how to spend the last moments with one’s terminally ill husband,”* reflecting a subtle shift from predominantly affirmative interpretations to more nuanced and ambivalent reflections. Furthermore, elements that had been prominent in the earlier interviews, such as the emphasis on surrounding support, appeared less salient in the follow-up narratives, suggesting a reorganization of narrative focus. Importantly, these developments were not apparent in the initial analysis and became discernible only through the longitudinal approach, making it possible to capture not only thematic continuity but also the gradual refinement and reconfiguration of meaning over time. Rather than representing a linear progression from negative to positive interpretations, the findings suggest a dynamic and evolving process in which previously constructed meanings are revisited, subtly rebalanced, and integrated within an ongoing life narrative. These findings highlight the presence of continuity and change while also revealing an underlying process through which meaning is gradually reorganized over time. Based on the findings, we propose a spiral model of meaning reconfiguration to conceptually integrate these temporal developments (Fig. 2). This model depicts how early meaning configurations characterized by relational affirmation, gratitude for caregiving, and emphasis on family cohesion are not replaced but revisited and refined through continued reflection. Over time, new experiential realities, such as confronting everyday life without one’s husband, introduce subtle tensions and prompt reinterpretation. Rather than overturning the previously derived meanings, this process appears to involve their integration within a broader and more complex life narrative. Importantly, the follow-up design made it possible to observe this gradual rebalancing of thematic salience. The emergence of ambivalence, refinement of caregiving meanings, and shifting emphasis from external support toward existential reflection suggest a dynamic restructuring rather than simple thematic addition. The spiral metaphor captures this process; meanings return but at a different level of integration shaped by lived time and future-oriented life repositioning. Therefore, this model conceptualizes bereavement meaning-making not as a linear progression toward resolution but as an ongoing and open-ended process in which prior interpretations are continuously elaborated and reorganized within the survivor’s evolving life context.

Below, we discuss how a wife who had initially viewed home-based end-of-life care for her husband in a positive light re-evaluated that experience two years later, and we examine the characteristics and significance of that shift.

### Reconstructing meaning over time

In this study, the initial interviews already revealed narratives oriented toward the future. Through the experience of caring for their husbands at home until the end of life, the wives came to recognize their own value and role, articulated as a “conviction in one’s own significance.” Even in the early period after bereavement, the wives had begun to ascribe meaning to their caregiving experiences within the broader context of their lives, reflecting on “how to live going forward” and, in some cases, taking concrete actions. These findings suggest that meaning-making and personal growth do not necessarily arise only with the passage of time after bereavement but may begin to take shape relatively early.

By contrast, the second interview was characterized not by the emergence of this future orientation, but by a more panoramic and integrated reconfiguration of meaning. With greater temporal distance, the wives revisited their husbands’ final days from a broader perspective and reinterpreted their husbands’ thoughts regarding the lives they left behind as sources of meaning that sustained their own present existence. Moreover, the aspirations expressed in the first interview—such as “wanting to be useful to others” and “wanting to make use of my experience”—were repositioned as more realistic and sustainable life choices.

While this positive meaning-making was maintained, additional narratives emerged over time that reflected a more distanced and reflective appraisal of the end-of-life caregiving process. In this context, participants began to voice feelings of regret and self-reproach, including statements such as “I distanced myself when faced with my husband’s death” and “I did not have enough emotional capacity to be considerate.”

This pattern accords with Neimeyer’s model of meaning reconstruction [10] and with Park’s concept of “integrative meaning-making” within posttraumatic growth (PTG) [12]. Whereas the first interview primarily reflected meaning generation, in which meaning and value were directly derived from the caregiving experience, the second interview illustrated a shift toward meaning integration, whereby these meanings were repositioned within the broader temporal trajectory of the participants’ lives. Importantly, the expressions of “regret” and “self-blame” observed in the second interview should not be interpreted as indicators of grief relapse or maladjustment. Rather, they appear to reflect a mature process in which participants, while maintaining a positive appraisal of their caregiving, became able to move beyond idealization or self-protective narratives and engage in a more realistic and holistic reassessment of their experiences. The coexistence of ambivalent emotions—such as confidence in having supported their husbands to the best of their ability alongside a wish that they could have done more—can thus be understood as a hallmark of advanced meaning reconstruction, deepening the personal significance of the loss and integrating it into the self-narrative.

Accordingly, the contribution of this study’s longitudinal findings lies not in determining whether participants began to contemplate their future lives, but in elucidating how an already present future orientation was reinterpreted, deepened, and stabilized within the self-narrative over time. These findings illuminate a process of maturation shaped by relationality and temporal distance among Japanese women who provided home-based end-of-life care for their spouses within a gendered cultural context.

### Reconstruction of lasting spousal bonds and connections

The theme of “the profound significance of existing as a couple” revealed in the first interview was reconfigured in the second interview as “the enduring bond of the married couple.” This theme contained two subthemes: *“a sense of fulfillment in the final days of daily life spent consciously as a couple”* and “*the sense of remaining connected to one's husband.*” The first subtheme involved the participant recounting the daily life spent at home with the husband during the terminal phase of the illness as “proof of having lived as a couple,” positively affirming the value of that time even after his death. Meanwhile, the second subtheme featured narratives expressing the feeling of “still living with my husband” even after the husband’s death, framing the relationship as a transformation, not a loss. This aligns with the concept of “continuing bonds” proposed by Klass et al. [13], illustrating a psychological process in which the relationship is not severed by bereavement but maintained and deepened in a new form. The theme of “the enduring bond of the married couple” corresponds to the final stage of the meaning reconstruction model proposed by Gillies and Neimeyer [11], which involves integrating the experience of loss into personal narrative. Moreover, given that Japanese wives positively accept the responsibility of caring for their husbands, with an internalized sense of duty to support them [24], the persistence of this bond may be understood as a culturally embedded meaning structure closely intertwined with gender role perceptions.

### Confronting life without a husband and deepening the relationship

The second interview revealed a new theme: confronting life without one’s husband. This psychological process emerged only with the passage of time after bereavement. This theme encompasses two subthemes: “*the sadness of life without one’s husband*” and “*sorting out one’s feelings*.” Within the “the sadness of life without one’s husband” subtheme, many participants described a persistent sense of loneliness, even as they became more accustomed to the husband’s absence. This indicates that grief is not a linear recovery process but a sustained one oscillating between loss and connection [25]. Additionally, this “sadness” is a form of “continuing bonds” [12] and can be understood as an internal relationship of “continuing to live with one’s husband.” Conversely, the subtheme of “sorting out one’s feelings” described the process of deepening self-understanding through communication with others. This “processing through narration” reflects the tendency in East Asian cultures to “organize emotions through relationships” [26]. This represents the reconfiguration of emotions mediated through relationality rather than introspection confined within the individual. Furthermore, while expressing gratitude for the help received from professionals, the wives reframed receiving support not as “weakness” but as “relational solidarity in shared caregiving.” This relational interpretation reflects the cultural characteristic of Japanese women to “organize emotions within relationships” [26].

Through their narratives, the wives redefined “the self that had cared for their husband,” finding new roles and meaning in life without the husband. This redefinition involved more than recovering from loss; it marked a process of rebuilding life on the foundation of the years lived with their husband, aligning with the “changed philosophy of life” dimension of posttraumatic growth (PTG) [27].

### From sustained bonds to relational growth

The themes of the enduring bond of the married couple and confronting life without the husband both aligned with the concept of continuing bonds [13], illustrating how the relationship with the husband persists in altered forms after death and deepens internally over time. Furthermore, although the wives positively framed the experience of caring for their husbands at the end of life as “pride in having supported their husbands,” they re-evaluated this meaning and re-articulated it within their post-bereavement lives, reconstructing a “self that continues to live with their husbands.” This can be understood as a narrative reorganization process within Neimeyer’s [10] “meaning reconstruction.”

Therefore, wives achieved relational growth through loss, not only growing as individuals but also developing their personalities through relationships with others and the deceased, under the cultural characteristic of shaping oneself through relationships. This aligns with the concept of “posttraumatic growth” [27] in PTG theory and corresponds with the Japanese cultural value of “forming the self within relationships” [26].

### Practical Implications: from the perspective of long-term, relational support by visiting nurses

This study’s findings indicate that wives who cared for their husbands with cancer at home until death experienced a reconstruction of their relationship with their spouse. They felt a sense of fulfillment in having supported their husbands until the end of life, yet they also carried lingering regret that “perhaps more could have been done.” This psychological process does not occur at the point of death but unfolds after bereavement. However, Japan’s medical and long-term care reimbursement frameworks restrict billable services to care delivered to the patient. As a result, bereavement follow-up for family members is not reimbursed, leaving its provision entirely to the organizational policies and capacities of individual home-visit nursing agencies. It is, therefore, important to recognize the practical value of the interactions that occur before death and during bereavement visits immediately afterward.

### Nature of support across phases

Consistent with the longitudinal patterns identified in this study, narrative-oriented support may need to change across different phases of the caregiving and bereavement trajectory.

Before death, support may involve explicitly recognizing the caregiver’s efforts, affirming caregiving as a shared relational process, and intentionally protecting moments of ordinary spousal interaction within the clinical context. Such relational acknowledgments may later provide an important foundation for meaning reconstruction, particularly among spouses who initially engage in positive appraisal of their caregiving experience. Beginning immediately after death, bereavement visits can function as structured yet flexible narrative spaces in which surviving spouses are invited to recount their experiences in their own words. When conducted by a nurse who was involved in the caregiving process and has established a trusting relationship with the family, this continuity may further facilitate meaning reconstruction. The nurse’s prior knowledge of the caregiving context and shared experiences can create a sense of relational safety that enables the bereaved spouse to revisit and reorganize meanings within an already established interpersonal framework. Approximately 80% of visiting nurse stations conduct bereavement visits following a patient’s death. Although this support is typically limited to a single contact [28], the encounter can nonetheless be significant for bereaved spouses. Even a brief interaction—particularly when grounded in an ongoing therapeutic relationship—may contribute to the early reorganization of meaning that continues over time. In the 6–24 months following bereavement, narrative support may focus on how caregivers interpret their caregiving experience and how they situate it within their broader life story while also remaining attentive to ambivalence or unresolved concerns. The present findings indicate that even at 33–46 months post-bereavement, reflective conversations that gently invite comparison between past and present perspectives may help some bereaved spouses recognize shifts in meaning and identity over time. However, such engagement should be tailored to individual readiness and not assumed to be universally appropriate.

### Practical constraints and early relational engagement

In the Japanese healthcare context, bereavement follow-up visits by home-visit nurses are typically not reimbursed under medical and long-term care insurance systems. Consequently, providing sustained long-term support to a bereaved spouse after the patient’s death may be challenging in routine practice.

Given these structural constraints, the findings suggest that relational support provided before death may hold particular importance for the bereaved spouse. Meaning-making processes continue long after bereavement, and therefore, interactions during end-of-life—such as acknowledging caregiving efforts, validating ambivalent feelings, and supporting authentic spousal connection—may serve as foundational experiences that can influence later grief trajectories.

In this sense, understanding the longitudinal process of bereavement does not necessarily imply prolonged post-death intervention. Rather, it underscores the significance of intentional relational engagement prior to death, which may indirectly support bereaved spouses even when formal follow-up is limited.

### Potential risks and ethical considerations

Narrative-oriented engagement may evoke emotional distress, particularly if unresolved regret, relational conflict, or ambivalence surfaces. Therefore, such conversations should always be voluntary for the bereaved spouses, responsive to their individual readiness, and conducted in a manner that prioritizes their psychological safety. Nurses should remain attentive to signs of overwhelming distress and be prepared to pause, redirect, or refer individuals to more specialized psychological or multidisciplinary services when needed. Importantly, the intention of these interactions is not to impose reinterpretation or promote positive reframing but to create space for self-directed reflection. When offered sensitively, flexibly, and with the consideration that meaning-making trajectories may vary among bereaved spouses, such encounters may enable gradual integrative meaning reconfiguration in some individuals.

### Limitations

This study has several limitations. First, all participants in the second interview were women, and the sample size was small (five individuals). Therefore, the changes in meaning construction demonstrated here represent an aspect rooted in the cultural and gendered backgrounds of female spouses. This makes it difficult to generalize the results. Future research should include comparative analyses from diverse perspectives, such as male spouses and other family members, to clarify gender differences and the influence of family relationships on meaning-making after bereavement.

Second, this study longitudinally examined changes in meaning-making over approximately four years, based on the first interview conducted six months to two years after bereavement and the second interview conducted approximately two years later. However, the length of time since bereavement varied among the participants, and the study was limited to comparisons between two time points. This imposes limitations on capturing the meaning reconstruction process in greater detail. Future research should employ multiwave tracking and incorporate a life story perspective to more comprehensively examine how loss experiences are integrated over time.

Third, participants were intentionally selected based on their prior articulation of relatively positive meaning-making. Therefore, the findings reflect longitudinal patterns within this subgroup and do not represent the full spectrum of bereavement experiences. Wives who continue to struggle with unresolved grief, familial conflict, or difficulty integrating the loss may exhibit different meaning reconstruction trajectories. This selection focus may have increased the visibility of affirmative reinterpretations over time. Future research should examine more diverse bereavement experiences, including those characterized by persistent distress or relational tension. Clinically, these findings suggest that while some bereaved spouses gradually reconfigure meaning in adaptive ways, others may require additional psychological and relational support to facilitate meaning integration. Support strategies should therefore remain sensitive to variability in meaning-making processes rather than assuming a universal trajectory of positive reconstruction.

Therefore, the present findings should not be interpreted as representing the general course of bereavement but rather as illustrating one possible pathway among those who initially demonstrated adaptive meaning reconstruction. To understand the diverse aspects of grief, future research should examine bereaved family members who experience difficulty in forming positive reinterpretations or in affirmatively viewing caregiving and end-of-life care.

## Conclusions

This study aimed to elucidate how wives who had previously derived positive meaning from caring for and witnessing the death of their husbands with cancer reinterpreted these experiences over time. The findings revealed that they continued to perceive an enduring bond with their husbands and reaffirmed the significance of their relationship as family members who shared the final phase of life. They also engaged in an ongoing process of reviewing and re-evaluating their caregiving experiences, thereby reconstructing the meaning of end-of-life care. Through third-party conversations, they organized their emotions, confronted life without their husbands, and deepened their personal understanding of life and death.

While many themes overlapped with those identified during the first interview (six months to two years post-bereavement), the present study newly identified the theme of *“confronting life without one’s husband,”* as well as an additional subtheme within *“the meaning attached to caring for one’s husband in the terminal phase,”* specifically *“regrets about how to spend the last moments with one’s terminally ill husband.”* These findings indicate that the meaning-making of caregiving and bereavement experiences is not static; rather, it is a dynamic psychological process that is continuously reconstructed over time following spousal loss.

## Supplementary Information

Supplementary Material 1.

## Data Availability

The data used in this study are available from the corresponding author upon request.

## Abbreviation

PTG: Posttraumatic growth
