# Supplementary material for: Meaning-making in home-based end-of-life care: a qualitative study of wives’ experiences after the loss of their husbands to cancer
Source: BMC Palliat Care. 2026 May 12;25:196. doi: 10.1186/s12904-026-02095-z (PMC13343870; doi:10.1186/s12904-026-02095-z)
Supplement: Supplementary file 1 — Supplementary Material 1. [file 12904_2026_2095_MOESM1_ESM.docx]

**Supplementary Table 1: Interview Guide for Bereaved Relatives of the Patient**

| **Sample Questions: Probes and Prompts** |
| --- |
| **1. Please provide details about yourself and your husband.**  1) Could you share your husband's medical diagnosis?  2) Could you indicate your husband's age at the time of his passing?  3) May we ask your age when your husband passed away?  4) Could you describe your family circumstances while you were caring for your husband at home? |
| **2. Please describe your caregiver approach and the support you received during the caregiving period from others.**  1) Kindly indicate the period during which you provided care for your husband at home.  2) Please explain the circumstances that led to the initiation of home care.  3) Were there any positive or meaningful experiences during the home care period? If so, please describe them.  4) During the home care process, did you encounter difficult, anxious, or burdensome experiences? If so, please elaborate.  5) Were there individuals who supported you while caring for your husband? If yes, please share specific words or actions that were particularly helpful. |
| **3. Please describe how the experience of caring for your spouse at home, and his passing there, has influenced your subsequent life.**  1) What personal meaning did your husband's home care hold for you?  (1) Looking back on your husband's home care, how do you feel about that experience now?  (2) Does that experience continue to provide support for you in the present? If so, what factors do you believe have influenced these feelings? Please provide precise details.  (3) Do you feel that these reflections are connected to what you shared during our previous interview? If so, please explain how they are related.  2) What personal meaning do you ascribe to the experience of caring for your husband at home during his terminal illness?   1. **Reflections on the caregiving experience**   (1) What meaning did you derive from the caregiving experience?   1. Looking back on the experience of caring for your husband at home until his passing, is there anything you feel you learned? If so, please share.   ・Do you think any particular events or thoughts are related to how you feel now? If so, please describe them in more detail.   1. What personal value do you feel the experience of caring for your husband at home held for you?   ・Do you believe specific events or reflections influenced how you feel now? If so, please explain.   1. **Reflections on family and social interactions after caregiving**   (2) If you found meaning in your interactions with family or others following the caregiving period, please share specific examples.   1. After caring for your husband at home, do you feel supported by your family or those around you now? If so, in what situations do you experience this support? 2. Having cared for your husband at home, what meaning does the connection you hold with family and others hold for you?   ・Do you feel your present thoughts or feelings are influenced by reflections you had after your caregiving experience? If so, please share what you believe has shaped these feelings.  3) Reflecting on your earlier insights, how do you now understand the meaning of caring for your husband with terminal cancer at home?  (1) Do you feel that the meaning you derived from caring for your husband at home has influenced or changed your thoughts and feelings in your personal life? If so, please describe these changes in detail. |
